# Targeted knockout of barley *Ycf54* demonstrates its essential function in the Mg-protoporphyrin IX monomethyl ester cyclase involved in chlorophyll biosynthesis

**DOI:** 10.1186/s41065-026-00693-8

**Published:** 2026-05-28

**Authors:** Helmy M. Youssef, Iris Hoffie, Otto Nordling, David Stuart, Shakhira Zakhrabekova, Radwa Y. Helmi, Jochen Kumlehn, Mats Hansson

**Affiliations:** 1 Faculty of Agriculture, Cairo University, Giza, 12613 Egypt; 2 Research, Development and Innovation Centre for Converging Science and Emerging Technology (RDI CoSET), Benha National University, P.O.11828, El- Obour City, Egypt; 3 Faculty of Natural Sciences III, Institute of Agricultural and Nutritional Sciences, Martin Luther University Halle-Wittenberg, Halle, 06120 Germany; 4 Leibniz Institute of Plant Genetics and Crop Plant Research (IPK), Corrensstr. 3, OT, Gatersleben, Seeland 06466 Germany; 5 Department of Biology, Lund University, Sölvegatan 35B, Lund, 22362 Sweden; 6 Genetics and Cytology Department, Biotechnology Research Institute, National Research Centre, Dokki, Giza, 12622 Egypt

**Keywords:** Barley, BchE, Chlorophyll deficiency, CRISPR, LCAA, MPE cyclase, Xantha

## Abstract

**Background:**

The Mg-protoporphyrin IX monomethyl ester cyclase is one of the 15 enzymes required for biosynthesis of chlorophyll in plants. The Ycf54 protein is a component associated with this enzyme and probably functions as a chaperone. Due to the lack of mutants in the *Ycf54* gene, we targeted this gene in barley (*Hordeum vulgare* L.) by RNA-guided Cas9 genome editing to dissect its role in chlorophyll biosynthesis.

**Results:**

Two guide RNAs were designed and delivered via Agrobacterium-mediated transformation. We generated four homozygous mutant alleles harboring deletions of 1, 2, 6 and 27 base pairs. The 27 bp deletion mutant also had a silent C-to-G point mutation before the deletion and a CC-to-AG mutation following the deletion changing a serine residue to glutamate. The mutants exhibited three distinct phenotypes: completely yellow leaves, green leaves with yellow stripes, and fully green leaves. Yellow-leafed plants were classified as Xantha mutants, characterized by an absence of chlorophyll but presence of carotenoids. The striped phenotype represented chimeric individuals with severe mutations in meristematic tissues. Yellow mutants carried homozygous 1 or 2 bp deletions, both of which caused translational reading frame shifts and premature truncation of the Ycf54 protein, likely disrupting chlorophyll biosynthesis and resulting in lethality. These mutations could only be stored in heterozygous lines. In contrast, the 6 and 27 bp deletions were in-frame and did not have any observed effect on the green phenotype, demonstrating that these mutations, and the CC-to-AG mutation, preserve Ycf54 functionality with maintained chlorophyll synthesis and plant viability.

**Conclusions:**

Previous studies have implicated Ycf54 in the folding and maturation of the cyclase enzyme. Our findings provide direct genetic evidence that *Ycf54* is essential for chlorophyll biosynthesis. Loss-of-function mutations result in a lethal, chlorophyll-deficient phenotype, underscoring the critical role of *Ycf54* in photosynthetic development.

**Supplementary Information:**

The online version contains supplementary material available at 10.1186/s41065-026-00693-8.

## Background

Chlorophyll molecules are among the most abundant light-harvesting pigments and essential for photosynthesis in plants. Chlorophylls are produced along with hemes and bilins via a branched tetrapyrrole biosynthetic pathway [1, 2]. The enzyme catalyzing the third step of the branch leading to chlorophyll, is named Mg-protoporphyrin IX monomethyl ester (MPE) cyclase. In the cyclase reaction, the isocyclic E ring is formed by insertion of oxygen as a ketone group at position 13^1^ and by attaching the 13^2^ carbon to the methene bridge between the C and D pyrrole rings, leading to the formation of protochlorophyllide [2]. In photosynthetic bacteria, with photosystems based on bacteriochlorophyll, the 13^1^ carbonyl group can be derived from water [3]. This anaerobic cyclase is encoded by *bchE* in, for example, *Rhodobacter sphaeroides* [3, 4]. In the aerobic reaction, one of the oxygen atoms of molecular oxygen is incorporated into the substrate and the other is reduced to water [3, 4]. Thus, the aerobic cyclase is an oxygenase whereas the anaerobic enzyme functions as a hydratase. The *bchE* gene was also found in *Rubrivivax gelatinosus* enabled to photosynthesize under various oxygen conditions [5]. In this organism, also the aerobic cyclase gene, *acsF*, was found. This made it possible to identifying the orthologous gene in other organisms, e.g. *Cth1* in *Chlamydomonas reinhardtii* [6, 7], *chlA1*, *chlA2*, *cycI* and *cycII* in *Synechocystis sp*. PCC 6803 [8, 9], *CHL27* in *Arabidopsis thaliana* [10] and *Xan-l* in barley (*Hordeum vulgare* L.) [11–13]. However, from studies based on fractionation of chloroplast extracts, it was obvious that several protein components are involved in the cyclase reaction both containing soluble and membrane-bound components [14]. This was further supported by the existence of barley *vir-k* mutants, which, like *xan-l* mutants, accumulate MPE upon feeding with the chlorophyll biosynthetic precursor 5-aminolevulinic acid [15, 16]. The *Vir-k* gene has now been identified and shown to encode a ferredoxin essential for cyclase activity [17].

The discovery of Hypothetical chloroplast open reading frame 54 (Ycf54) in *Synechocystis* [18] and Low Chlorophyll Accumulation A (LCAA) in tobacco (*Nicotiana tabacum* L.) [19], identified a third protein associated with the cyclase enzyme in oxygenic phototrophs. Ycf54 was found to be essential for enzymatic activity of Arabidopsis CHL27 and *C. reinhardtii* Crd1 when expressed in *R. gelatinous* and *Escherichia coli* (Chen and Hunter 2020). Further, production of recombinant barley XanL in *E. coli* required co-expression of *Ycf54* and the function of Ycf54 was suggested to concern correct folding and maturation of XanL rather than participation in the enzymatic reaction [12]. This supported a role for Ycf54 as a chaperone; however, its essentiality could not be determined.

Since the discovery of the clustered regularly interspaced short palindromic repeat (CRISPR)-associated (Cas)9 endonuclease system, which can be used as programmable tools for genome editing, its application has revolutionized many research fields including plant science [20]. By using CRISPR/Cas-derived tools, single or multiple mutations can be created at a target gene without altering the genetic background, allowing powerful genetic analyses of individual mutants. Nowadays, in barley, wheat (*Triticum aestivum*) and other cereal crops, the Cas9 system is the molecular tool of choice for targeted mutagenesis [21, 22]. The use of such gene editing tools was previously reported in barley [23], wheat [24, 25], maize (*Zea mays*) [26, 27] and rice (*Oryza sativa*) [28, 29] to improve plant resistance to biotic stresses and tolerance of abiotic stresses and to increase yield potential. Using Cas9-mediated genome editing in barley involving single guide RNAs targeting *HvARE1*, that is expressed in leaves, shoots and roots under low nitrogen conditions, 22 M_1_ plants were generated, from which four M_2_ families harbored missense and/or frameshift mutations based on genotyping. Homozygous mutant *are1* lines showed a rise in growing traits such as plant height, tiller number and yield. Moreover, in the grain filling stage, these lines presented a 1.5- to 2.8‐fold increase in total chlorophyll content in the flag leaf compared to the genetic background cultivar Golden Promise [30]. In another study, the *HvPM19* gene encoding a plasma membrane protein regulating grain dormancy in barley was targeted using Cas9. The results showed that the mutant lines confirmed the possibility of genome editing in creating the desired mutations and stable transmission of mutations to subsequent generations [31]. Thus, Cas9 can be used for scientific purposes or to improve agronomic traits in barley.

In the present study, we targeted the barley *Ycf54* gene by Cas9-mediated gene editing to address its role in the cyclase reaction. Mutants were obtained which displayed a yellow Xantha phenotype. The mutants were lethal beyond the seedling stage clearly demonstrating the essential function of *Ycf54* in chlorophyll biosynthesis. The study complements previous studies on the barley cyclase, which have been based on mutants in the *Xan-l* and *Vir-k* genes [11, 17].

## Methods

### Phylogenetic analysis

The barley cDNA sequence was deduced from the *Ycf54* gene HORVU.MOREX.r3.4HG0379270 using the barley genome explorer (BARLEX) (https://apex.ipk-gatersleben.de/apex/f?p=284:10::::::). cDNA sequences of 26 *Ycf54* orthologues were identified from different plant species, *Synechocystis* sp. PCC6803 and *Chlamydomonas reinhardtii* (Table S2). A multiple sequence alignment was performed using Multiple Sequence Alignment (MSA) (Clustal Omega: https://www.ebi.ac.uk/jdispatcher/msa/clustalo [32]. The aligned sequences were imported into MEGA X for phylogenetic analysis. The best-fit evolutionary model was determined using the model selection tool implemented in MEGA. A phylogenetic tree was constructed using the Neighbor-Joining method with 1000 bootstrap replicates to assess branch support [33].

### Generation of *ycf54* mutants by genome editing

Two gRNAs were used in tandem to target barley *Ycf54* (HORVU.MOREX.r3.4HG0379270). The online tools WU-CRISPR (https://crisprdb.org/wu-crispr-website/index.html*)* [34, 35] and RNAfold (http://rna.tbi.univie.ac.at/cgi-bin/RNAWebSuite/RNAfold.cgi*)* were used to select effective target motifs as described in [20]. The constructs were generated using the CasCADE modular vector system, which is based on a hierarchical Golden Gate cloning strategy [36]. Oligos (Table S1) with complementary target sequences and overhangs for cloning into BsaI restriction sites were annealed and cloned into BsaI–linearized vectors pIK1, pIK3, pIK6 and pIK8 containing either the *OsU3* or the *TaU6* promoter. The resulting four vectors (pHY1-4) containing four gRNA units were digested with Esp3I and assembled into the pIK19 vector, generating vector pHY5. The gRNA expression units were combined with a maize codon-optimized SpCas9 expression unit (pIK83) using the enzyme BsaI to generate pHY6.

### Plant transformation and cultivation

The binary vector was electroporated (Gene Pulser, Bio-Rad) into *Agrobacterium tumefaciens* strain AGL1 [37] and DNA transfer into immature embryos of spring barley Golden Promise was performed [38]. Immature embryos were excised from caryopses 12–16 days after pollination and cultivated with Agrobacterium strain AGL1 carrying the respective vector for 48–72 h. Then, the explants were cultivated for callus induction under selective conditions using timentin and hygromycin, followed by plant regeneration.

### Plant material and growing conditions

Two-rowed spring-type barley cultivar Golden Promise was used in this study. The donor material and regenerated M_1_ and M_2_ plants were cultivated in the greenhouse at IPK-Gatersleben, Germany as described previously [39]. M_3_ plants and plants used for crosses were grown in the greenhouse of Department of Biology, Lund University, Sweden according to [38]. For segregation analyses, full spikes were planted in vermiculite and grown on the lab bench for ten days. Crosses between plants segregating from heterozygous parents and Golden Promise were performed as described in [40].

### Genomic DNA extraction, PCR and sequencing of target locus

Genomic DNA was extracted from seedling leaves grown for 1–2 weeks. A phenol/chloroform/isoamyl alcohol (26:24:1; AppliChem GmbH, Darmstadt, Germany)-based method [41] was used for DNA extraction from the samples collected from M_1_ and M_2_ plants at IPK-Gatersleben, Germany. DNA extraction from samples collected from M_3_ plants was done using REDExtract-N-Amp™ Plant PCR Kit (Sigma-Aldrich) or the DNeasy Plant Mini Kit (Qiagen) at Department of Biology, Lund University, Sweden.

Primers producing amplicons covering the genomic target locus were used for PCR (Table S1). Purified PCR products were sent for Sanger sequencing (LGC Genomics) and resulting sequences were analyzed using the Unipro UGENE software. The transgenic status of the plants was determined by PCR amplification of the *cas9* and *hpt* genes (Table S1). 

### Chlorophyll measurements

Measurements of chlorophyll content in seedling leaves were done using a Hansatech Instruments CL-01 Chlorophyll Meter (Hansatech Instruments Ltd., King’s Lynn, UK). Twenty seedling leaves were measured. Maximum quantum efficiency of photosystem II (Fv/Fm) was measured with an Imaging-PAM MAXI (Heinz Walz GmbH, Germany). Significant differences were calculated using a two-sided Student’s t test.

### Modeling of a Ycf54 3D structure

AlphaFold3 [42] (www.alphafoldserver.com) was used to construct a 3D structural model of the *Ycf54* gene product without the chloroplast transit peptide. The chloroplast transit peptide was predicted using Target P (https://services.healthtech.dtu.dk/services/TargetP-2.0/).

### Immunoblots analysis

Polyclonal rabbit antibodies raised against barley Ycf54 were generated as described previously [12]. Total protein was extracted as described by [17]. SDS-polyacrylamide gel (PAGE) and immunoblots were performed as described previously [12].

## Results

### Targeted knockout of barley *Ycf54*

**Fig. 1:**
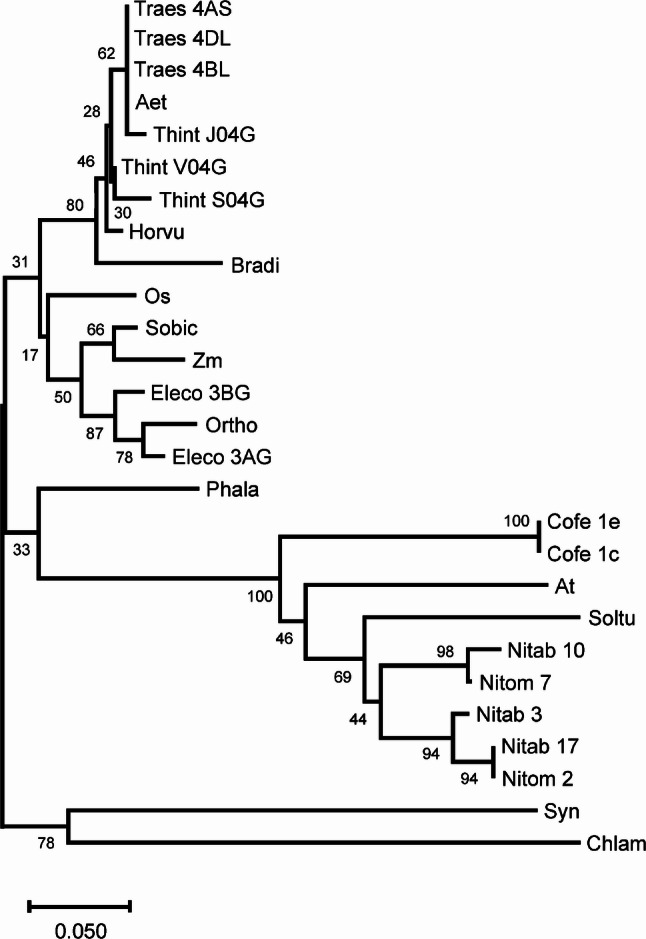
Phylogenetic tree of *Ycf54* cDNA sequences from diverse photosynthetic organisms, constructed using MEGA X. A total of 27 cDNA sequences were analyzed. Detailed accession information for all sequences is provided in Table S2. Branch lengths represent nucleotide substitutions per site (scale bar = 0.050). Bootstrap support values (%) were calculated from 1000 replicates and are shown at each node

Analysis of the barley genome sequence identified a single *Ycf54* gene (HORVU.MOREX.r3.4HG0379270). The barley *Ycf54* cDNA sequence was subsequently used to identify orthologous genes in a representative set of other photosynthetic species (Table S2). In most cases, the number of *Ycf54* gene copies corresponded to the ploidy level of the respective species. The identified sequences were used to construct a phylogenetic tree, which resolved several distinct clades (Fig. 1). Barley *Ycf54* clustered with orthologues from other cereals and grouped closely with members of the grass family. Our comparative analysis indicates that *Ycf54* is widely conserved across photosynthetic plant species. Although our previous studies demonstrated that *Ycf54* performs an important biological function, they did not establish whether the gene is essential [12]. Therefore, we sought to generate a barley *Ycf54* loss-of-function mutant to directly assess its functional significance in plants.

**Fig. 2:**
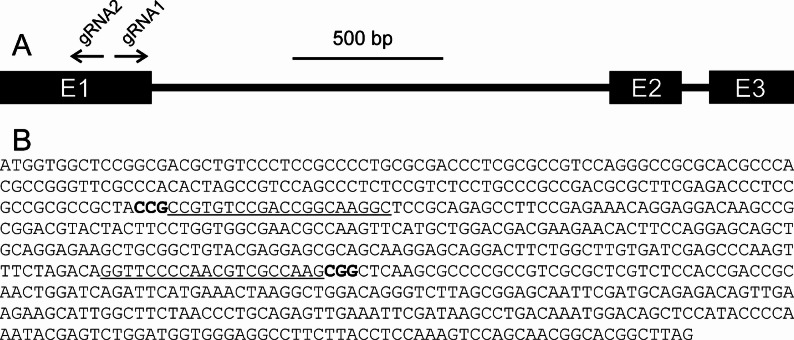
Barley *Ycf54* and the target motifs of used gRNAs. **A**: The *Ycf54* gene is located on chromosome 4 H and includes three exons. Two gRNAs were constructed according to the sequence of the first exons. **B**: The *Ycf54* cDNA sequence and the target motif sequences of gRNA2 and gRNA1 underlined. Protospacer-adjacent motif (PAM) in bold

**Fig. 3:**
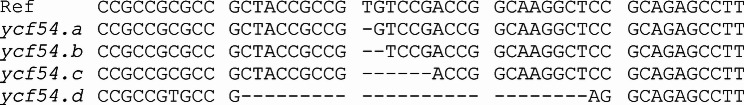
The obtained mutations compared to the barley reference sequence (Ref). Mutants *ycf54.a*,* ycf54.b* and *ycf54.c* have a single nucleotide deletion in position 161, a two-bp deletion in position 161–162, and a six-bp deletion in position 161–166, respectively. Mutant *ycf54.d* has a deletion of 27 nucleotides in position 152–178, as well as a C-to-T point mutation at position 147 and a CC-to-AG mutation at positions 179–180

To induce loss-of-function mutations in the *Ycf54* gene, two different target motifs were selected within the first exon; one forward and one reverse (Fig. 2). Two guide RNAs (gRNAs) were designed accordingly and fused into the plasmid p6i-d35S-TE9. Using this binary vector, immature embryos from barley cultivar Golden Promise were transformed via Agrobacterium-mediated DNA transfer, which resulted in eight primary transgenic plants. DNA sequence analysis of PCR products from Golden Promise and these plants showed that five of the transgenic plants had mutations in the target region of gRNA2. The M_1_ plants BG898E1, BG898E3 and BG898E8 were selected and proceeded to the M_2_ generation. Thirty M_2_ grains were planted and resulting M_2_ plants were genotypically and phenotypically analyzed. DNA sequence analyses showed segregation of different mutant alleles in each of the lines. In BG898E1, ten M_2_ progeny contained a one-bp nucleotide (T) deletion (-1) at position 161 relative to the ATG start codon, six plants carried a six-nucleotide (TGTCCG) deletion (-6) at position 161–166, and 12 plants were found to be heterozygous/chimeric (Fig. 3). Eight M_2_ progeny from plant BG898E3 had the same -1 mutation, while 16 plants were heterozygous/chimeric. From the third transgenic plant BG898E8, only 14 progenies germinated. These 14 plants carried two different mutant alleles. Seven plants had a two-nucleotide (TG) deletion (-2) at positions 161–162 and seven plants showed a 27-nucleotide (CTACCGCCGTGTCCGACCGGCAAGGCT) deletion at position 152–178 combined with a C-to-T point mutation at position 147 and a CC-to-AG mutation at positions 179–180 (-27) (Fig. 3).

### Phenotypic effects of the mutations

**Fig. 4:**
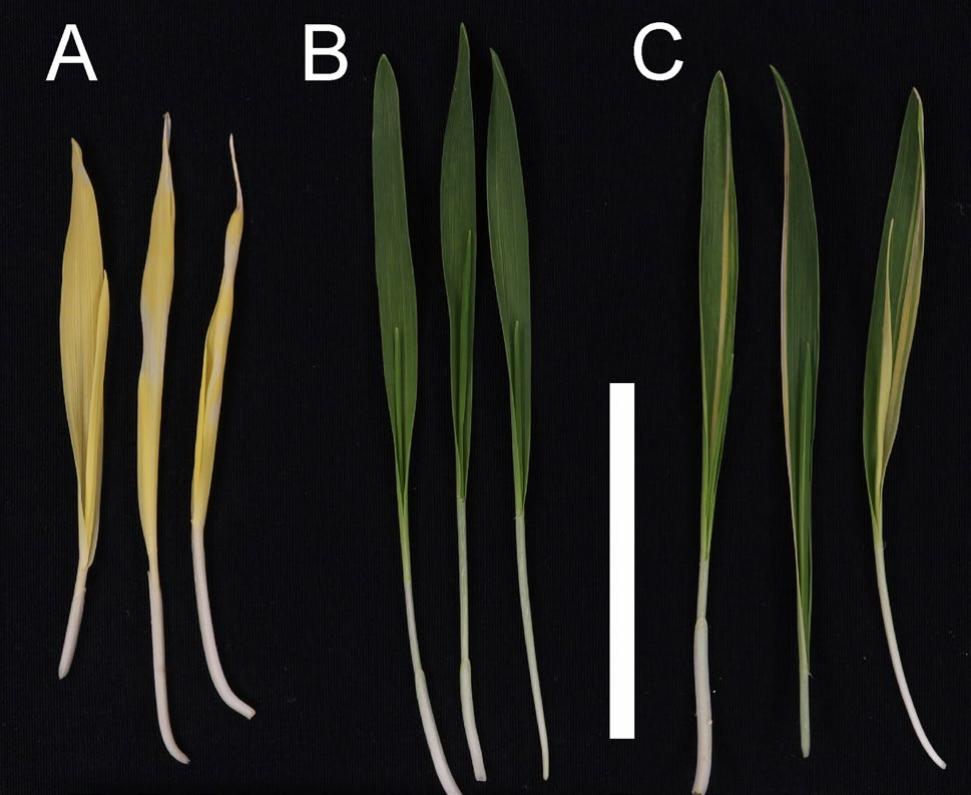
Three different phenotypes of barley *ycf54* mutant plants. Scale bars correspond to 5 cm. **A**: Fully yellow leaves. **B**: Fully green leaves. **C**: Green leaves with yellow stripes

**Fig. 5:**
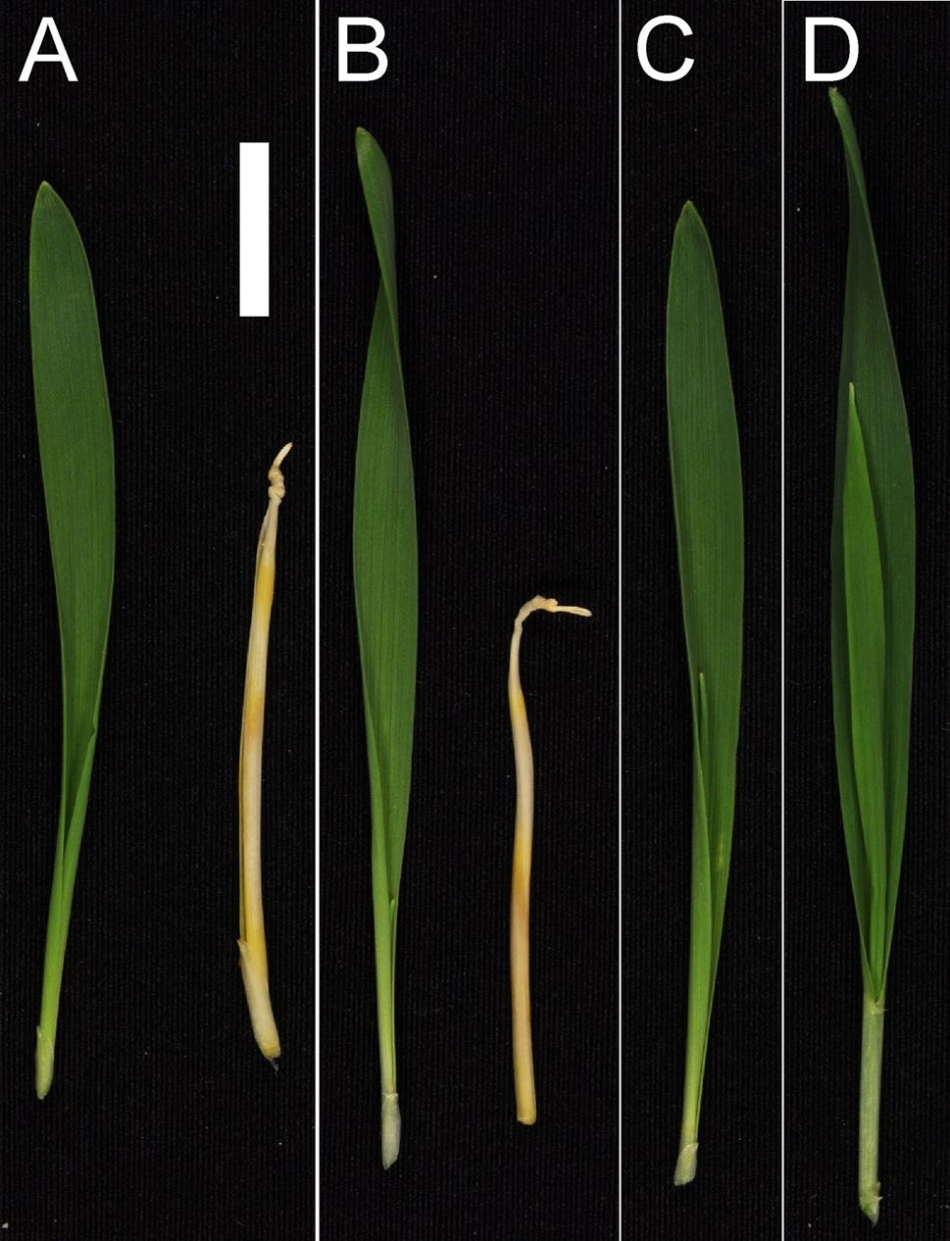
Segregation of F_2_ plants from crosses between the different Cas9-triggered *ycf54* mutants and Golden Promise. **A** and **B**: Segregating plants from mutants *ycf54.a* and *ycf54.b* harboring a single nucleotide deletion and a two-bp deletion, respectively. **C** and **D**: F_2_ seedlings from mutants *ycf54.c* and *ycf54.d*, respectively, showing no phenotypic segregation

 Three distinct phenotypes were observed in the M_2_ and M_3_ generations of the mutants. The first group had completely yellow leaves characteristic of barley homozygous Xantha mutants, which can synthesize carotenoids but not chlorophyll and therefore are lethal beyond the seedling stage. The yellow *ycf54* mutants carried the -1 or -2 mutations in homozygous form (Fig. 4A). We noted that the yellow plants had necrotic segments when grown in light-dark cycles, which make them also resemble barley Tigrina mutants [43]. The second group, with apparently fully green leaves, included plants that were heterozygous for any of the mutations or carried the -6 or -27 mutations in homozygous form (Fig. 4B). The third group had longitudinal green and yellow stripes (Fig. 4C). These plants were chimeric and DNA sequencing indicated presence of the -1 or the -2 deletion. It was noted that all chimeric plants and many of the other M_2_ plants contained the hygromycin resistant gene, i.e. they were likely to carry the *Cas9* and gRNA transgenes as well. In order to obtain stable mutant lines, we performed crosses between cultivar Golden Promise and M_3_ plants derived from BG898E1, BG898E3 and BG898E8 lines. F_1_ grains were planted and F_1_ leaf material was genotyped for the hygromycin resistance gene. F_1_ plants with absence of the hygromycin resistance gene were selected and their F_2_ grains were collected. Segregation for the yellow Xantha phenotype were studied in the F_2_ generation by germination of 30 F_2_ grains. Mutants carrying the -1 or -2 deletion, but not the -6 or -27 mutation, segregated into yellow and green seedling leaves (Fig. 5). One line of each type was selected and saved as reference line and named *ycf54.a* (carrying the -1 deletion in heterozygous form), *ycf54.b* (carrying the -2 deletion in heterozygous form), *ycf54.c* (homozygous for the -6 deletion) and *ycf54.d* (homozygous for the -27 deletion combined with the C-to-T point mutation at position 147 and the CC-to-AG mutation at positions 179–180). The amount of chlorophyll in Golden Promise and F3 plants of *ycf54.c* and *ycf54.d*, and the segregating plants of *ycf54.a* and *ycf54.b*, was quantified. No differences were seen between green leaves of the four mutants and the cultivar Golden promise. Statistic significant differences were only seen when the yellow plants, homozygous for the -1 and -2 deletions, were compared to green plants (Table S3). Since no apparent phenotypic effects with respect to chlorophyll content could be seen in green homozygous *ycf54.c* and *ycf54.d* plants compared to Golden Promise, we analyzed leaves for possible photosynthetic damages. The maximum quantum efficiency of photosystem II (Fv/Fm) of *ycf54.c*, *ycf54.d* and cultivar Golden Promise were not significantly different from each other (0.753 ± 0.013, 0.752 ± 0.011 and 0.755 ± 0.0096, respectively).

### Effect of the mutant alleles on the Ycf54 protein

**Fig. 6:**
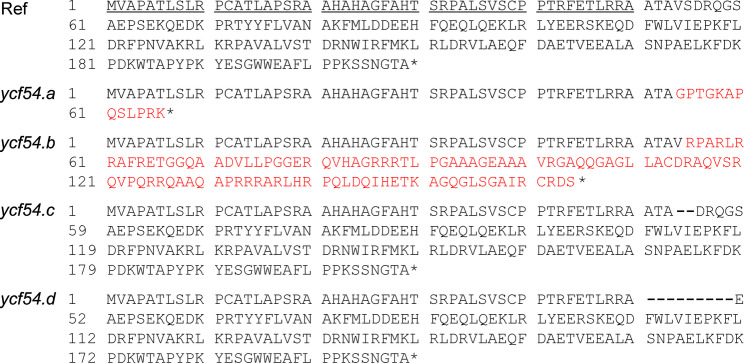
The protein sequences deduced from the *Ycf54* gene (Ref) and the four different *ycf54* mutants. The first 50 amino-acid residues were predicted to constitute the transit peptide (underlined in the Ref sequence). Residues indicated by red letters are results of the -1 and -2 out-of-frame deletions in *ycf54.a* and *ycf54.b*, respectively. Dashes indicate absent residues due to the -6 and -27 mutations in *ycf54.c* and *ycf54.d*, respectively. In the latter, the removed residues are directly followed by a Ser-to-Glu substitution

**Fig. 7:**
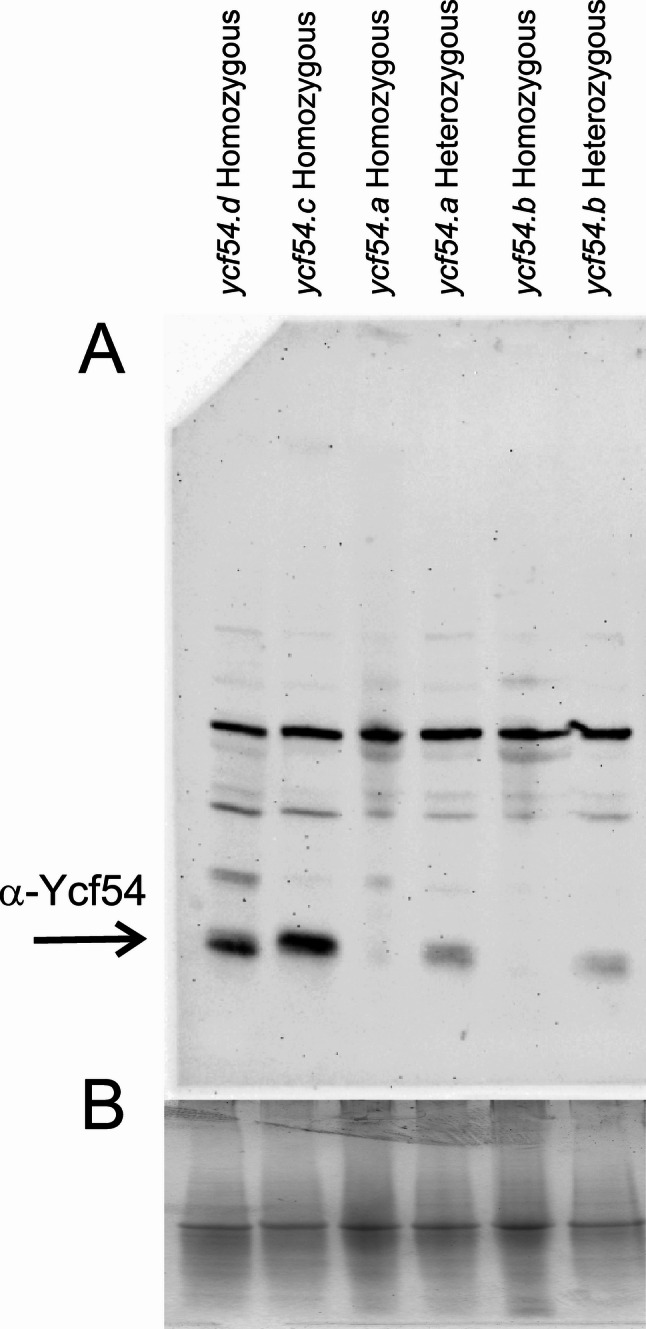
Presence of Ycf54 protein in segregating Cas9-generated barley *ycf54* mutants by immunoblot analysis. **A**: Ycf54 protein is absent in the yellow homozygous mutants *ycf54.a* and *ycf54.b* with one- and two-nucleotide deletions, respectively. In contrast, Ycf54 is present in the green homozygous mutants of *ycf54.c* and *ycf54.d*, as well as in heterozygous mutants of *ycf54.a* and *ycf54.b*. The predicted molecular mass of Ycf54 is 24 kDa. **B**: A Coomassie-stained replica gel was run as a loading control. The Coomassie-stained image has been compressed

The introduced mutations are all located in the 5’-region of exon one of the *Ycf54* gene. The single nucleotide deletion in *ycf54.a* led to a frame shift and eventually a premature stop codon. The resulting protein consisted of 53 native amino-acid residues followed by 13 non-native residues (Fig. 6). Similarly, the two-bp deletion in *ycf54.b* also caused a frameshift but resulted in a polypeptide of 54 native amino-acid residues and 110 non-native residues. The deletion of six nucleotides in *ycf54.c* was an in-frame deletion, which removed two amino-acid residues from the protein. In *ycf54.d*, the deletion of 27 nucleotides removed nine amino-acid residues directly followed by a Ser-to-Glu substitution due to the additional CC-to-AG mutation. The 50 first amino-acid residues were predicted to form the chloroplast transit peptide. Thus, all mutations affect the protein in the very beginning of the mature protein, which does not have a defined structure according to an AlphaFold3-generated structural model (Figure S1). Western blot analyses revealed that the out-of-frame deletions in *ycf54.a* and *ycf54.b* cause absence of the Ycf54 protein in yellow seedling tissues (Fig.  7).

## Discussion

 Barley is an important crop plant and during the last 90 years, many mutants have been induced by physical (e.g. x-ray and neutron irradiation) or chemical (e.g. ethyl methanesulphonate and sodium azide) treatment in breeding programs [43]. Some mutations have become established in today’s elite cultivars like the *sdw1.d* mutation, which causes deficiency in gibberellic acid biosynthesis and provides lodging resistance due to a short and sturdy culm [44]. However, most mutations had too strong negative effects on yield. Still, they are of scientific interest since they can be studied to reveal molecular processes in plants. Chlorophyll mutants were among the first mutants to be isolated since they are obvious already at the seedling stage. Therefore, they were used to optimize the mutagenic procedures. We have previously used barley *xan-l* and *vir-k* mutants to learn about the cyclase enzyme of the chlorophyll biosynthetic pathway [11, 17]. These mutants were isolated between 1954 and 1975 [17]. In addition to the *Xan-l* and *Vir-k* gene products, other proteins like Ycf54 have also been shown to be important for the cyclase reaction. Since it is not known whether any *ycf54* mutations are available in existing barley mutant collections, we explored Cas9-mediated gene editing to obtain *ycf54* mutant lines. Four stable mutants were obtained out of eight regenerated plants, which is consistent with previous findings in targeted mutagenesis in barley [22]. Two in-frame deletion mutants had no apparent effect of the phenotype. However, two out-of-frame deletion mutants demonstrated that plants homozygous, but not heterozygous, for these mutations have a yellow Xantha phenotype and are devoid of chlorophyll. This phenotype is shared with knockout *xan-l* mutants and demonstrates that the *Ycf54* gene is as important as *Xan-l* for the cyclase reaction. Mutants deficient in *Vir-k* show trace amounts of chlorophyll, which suggests that other ferredoxins can provide electrons to the reaction. Still *vir-k* mutations are lethal demonstrating that transfer of electrons by other ferredoxins is very limited, which also makes the *Vir-k* gene product an essential component for the cyclase reaction [17].

The *Xan-l* gene product is a monooxygenase and the core protein of the cyclase enzyme. The cyclase remains as one of the least studied enzymes of the chlorophyll biosynthetic pathway. Recombinant expression of *Xan-l* and its homologs from other species in *Escherichia coli* has only been successful when combined with expression of *Ycf54* [12, 45]. Possibly, Ycf54 is required for proper folding and maturation of XanL. In the present study, we demonstrated that Ycf54 plays a critical and essential role in chloroplast function, as knockout mutations involving 1 and 2 bp deletions of the gene resulted in lethal phenotypes due to a complete disruption of chlorophyll biosynthesis. These mutations lead to the generation of premature stop codons, causing truncated Ycf54 proteins that are unable to participate in the chlorophyll biosynthesis pathway. This finding aligns with previous studies in rice, which have shown that Ycf54 is crucial for maintaining chloroplast integrity and function [46]. A non-functional Ycf54 disrupts the chlorophyll biosynthesis pathway, ultimately leading to inability to produce chlorophyll, a vital component for photosynthesis, resulting in plant lethality. In contrast, a small amount of chlorophyll could be detected in *Synechocystis* strains in which the *Ycf54* gene had been deleted [47]. Interestingly, accessory proteins connected to various binuclear iron monooxygenases are common although they do not show obvious sequence similarities. It was reported that the propane monooxygenase encoded by *mimABCD* in *Mycobacterium goodie* required co-expression of *mimG* to be obtained in active form [48]. The *mimG* gene product is similar to the chaperonin GroEL. In contrast, recombinant production of a phenol hydroxylase of *Pseudomonas* sp. OX1 was not dependent on co-expression of its accessory protein PHK [49]. Instead, it was involved in increasing the affinity of the hydroxylase for iron. This work further supports the understanding that Ycf54 is indispensable for chloroplast function across different plant species. We conclude that further analyses are required to understand the biochemical mechanism of the essential Ycf54 component of the cyclase reaction.

## Conclusions

In conclusion, the Mg-protoporphyrin IX monomethyl ester cyclase, a crucial enzyme involved in chlorophyll biosynthesis in plants, requires the Ycf54 protein for its proper function. To explore the role of Ycf54 in chlorophyll biosynthesis, we targeted the *Ycf54* gene in barley using RNA-guided Cas9 technology. Four distinct homozygous mutant alleles were successfully obtained. Phenotypic analysis of these mutants revealed three distinct leaf color patterns: fully yellow, green with yellow stripes, and fully green. The yellow-leafed plants exhibited an absence of chlorophyll but presence of carotenoids. These plants harbored homozygous 1 and 2 bp deletions, indicating that the mutations were recessive and lethal. These mutant lines were named *ycf54.a* and *ycf54.b*, respectively. In contrast, mutant lines named *ycf54.c* and *ycf54.d* harboring 6 and 27 bp in-frame deletions, respectively, did not affect the plant phenotype, and these plants remained fully viable and green. Mutant *ycf54.d* carrying the 27 bp deletion also had a silent C-to-T point mutation at position 147 and a CC-to-AG mutation at positions 179–180 resulting in a Ser-to-Glu substitution. Apparently, the Ser-to-Glu substitution had no obvious effect on the plant phenotype. Overall, our results provide compelling evidence that the Ycf54 protein is indispensable for chlorophyll biosynthesis in plants. The identification of these mutations in barley underscores the critical role of Ycf54 and opens for further studies into chlorophyll production and the broader implications of chloroplast biogenesis.

## Supplementary Information

Supplementary Material 1: Fig S1. AlphaFold3 generated structural model of *Ycf54*.

Supplementary Material 2: Table S1. Primer sequences used in the study. Table S2. *Ycf54* cDNA sequences used for phylogenetic analysis. Table S3. Chlorophyll content in seedling leaves of segregating *ycf54* mutants.

## Data Availability

Data are contained within the article.
