# Supplementary material for: Targeted knockout of barley Ycf54 demonstrates its essential function in the Mg-protoporphyrin IX monomethyl ester cyclase involved in chlorophyll biosynthesis
Source: Hereditas. 2026 May 28;163:66. doi: 10.1186/s41065-026-00693-8 (PMC13221752; doi:10.1186/s41065-026-00693-8)
Supplement: Supplementary file 1 — Supplementary Material 1: Fig S1. AlphaFold3 generated structural model of Ycf54. [file 41065_2026_693_MOESM1_ESM.docx]

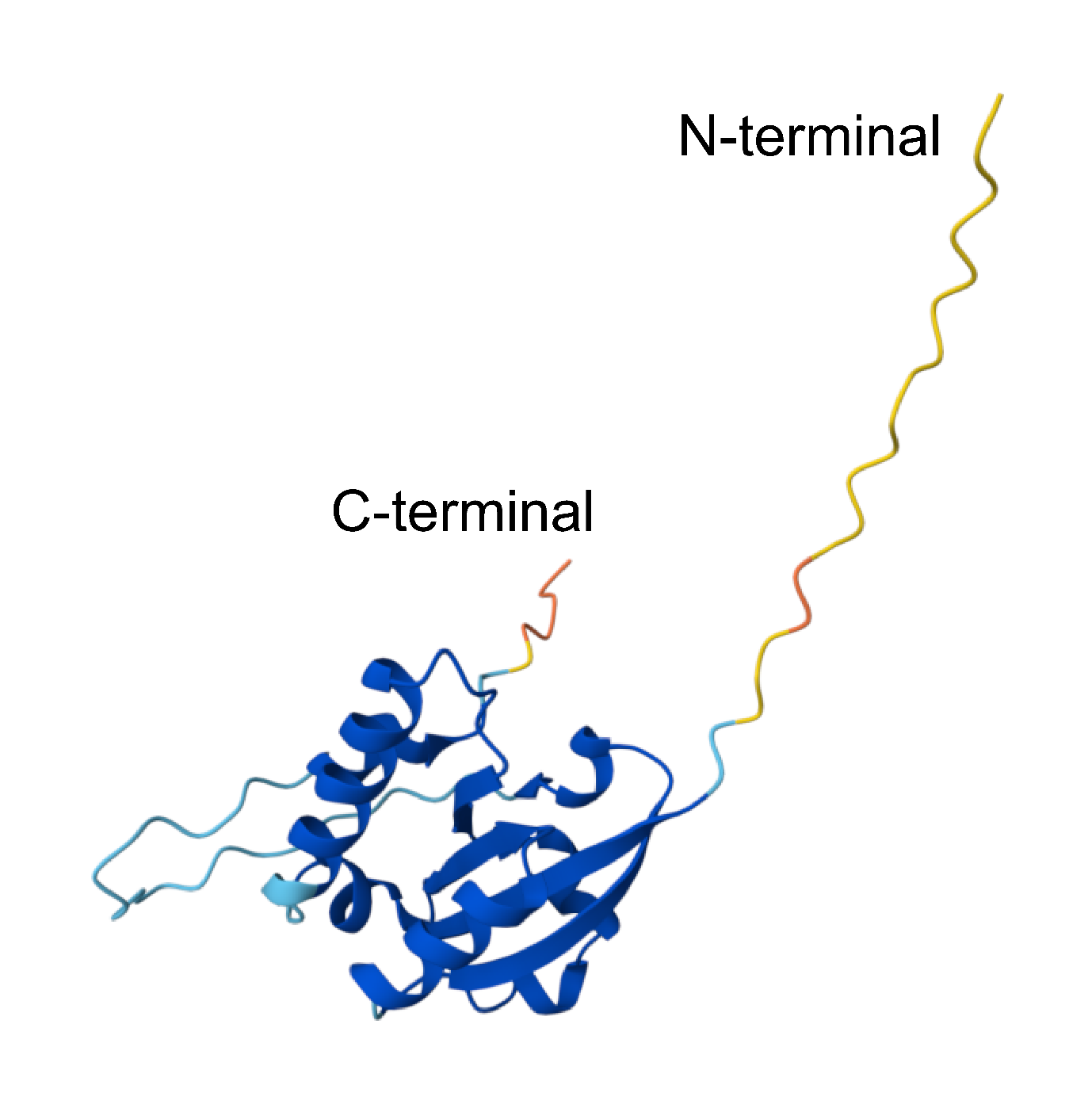


**Fig. S1** AlphaFold3 structural model of the mature polypeptide (residues 51-159) of barley Ycf54. The 20 N-terminal and five C-terminal amino-acid residues (yellow and orange color) are not predicted to have a defined structure.
